# Bio-optical characterization of selected cyanobacteria strains present in marine and freshwater ecosystems

**DOI:** 10.1007/s10811-015-0774-3

**Published:** 2016-01-14

**Authors:** Bożena Wojtasiewicz, Joanna Stoń-Egiert

**Affiliations:** Department of Physical Oceanography, Institute of Oceanography, University of Gdańsk, Marszałka Piłsudskiego 46, 81-378 Gdynia, Poland; CSIRO Oceans and Atmosphere, Floreat, WA Australia; Marine Physics Department Polish Academy of Sciences, Powstańców Warszawy 55, 81-712 Sopot, Poland

**Keywords:** Cyanobacteria, Chlorophyll, Carotenoids, Phycobilins, Absorption, Size structure

## Abstract

The optical properties, i.e., absorption and scattering spectra of ten strains of cyanobacteria from the Baltic Sea and Pomeranian lakes (*Aphanizomenon flos-aquae* KAC 15, *Microcystis aeruginosa* CCNP 1101, *Anabaena* sp. CCNP 1406, *Synechocystis salina* CCNP 1104, *Phormidium* sp. CCNP 1317, *Nodularia spumigena* CCNP 1401, *Synechococcus* sp. CCNP 1108, *Nostoc* sp. CCNP 1411, *Cyanobacterium* sp. CCNP 1105, *Pseudanabaena cf. galeata* CCNP 1312) grown under low light conditions were investigated. Moreover, the chlorophylls, carotenoids, and phycobilin composition as well as the size structure of chosen cyanobacteria were measured. Studied species revealed high diversity both in optical properties with the absorption spectra similarity index ranging from 0.67 to 0.94 and the pigment composition. The chlorophyll-specific absorption coefficient at 440 nm *a*_ph_*(440) varied between 0.017 and 0.065 m^2^ mg^−1^. The influence of the package effect was only observed in the case of large filamentous cyanobacteria like *N. spumigena* or *Nostoc* sp. Interestingly, the package effect factor *Q*_a_*(675) for large-celled *Anabaena* sp. was 0.92. Besides chlorophyll *a*, only echinenone, *β*-carotene, and phycocyanin were present in all analyzed cyanobacteria strains. Zeaxanthin, which is widely used as a marker pigment for cyanobacteria, was absent in the toxic *N. spumigena* and *Anabaena* sp., which are the species that occur in the Baltic Sea most frequently causing summer cyanobacterial blooms. The investigation also showed that the sample preservation technique can introduce some major errors within the absorption band affected by the phycocyanin absorption.

## Introduction

The energy from the sun reaching the Earth’s surface is the main source of energy which is absorbed by sea water. It has been estimated that even more than 90 % of the solar energy at the sea surface is absorbed and transformed into different kinds of internal energy necessary for various types of processes (e.g., Woźniak and Dera 2007). Among them, the photosynthesis and water heating seem to be the most important from the point of view of their consequences in the whole ecosystems. The knowledge on the absorptive properties of seawater, especially within the visible band, seems to be crucial for many studies on marine environment, like modeling the solar energy transport within the water column (Mobley et al. 1993; Mobley 1994; Chami et al. 2001), modeling the productivity of water reservoirs (e.g., Sosik 1996), or in various kinds of remote sensing algorithms (Stramska et al. 2003; Woźniak 2014).

The optically active seawater constituents are water molecules, colored dissolved organic matter (CDOM), and particulates, including living phytoplankton. In the Baltic Sea, the main bloom-forming organisms in summer are cyanobacteria also called blue-green algae. They are prokaryotic organisms capable to photosynthesize and characterized by both bacterial and plant features. Cyanobacteria can exist as filaments, single cells, and colonies. Some of the species are capable of atmospheric nitrogen fixation. The antennae system of the photosynthetic apparatus in all cyanobacteria, besides carotenoids, is formed by complexes of various biliproteins called phycobilisomes located in the membranes of the thylakoids (e.g., MacColl 1998). The most commonly occurring phycobilins that absorb light within the band from 450 to 660 nm are phycoerythrin (PE), phycocyanin (PC), and allophycocyanin (AP) (e.g., Grossman et al. 1993). The presence of various types of pigments whose concentration is highly variable and dependent on the environmental conditions is the main factor influencing the spectra of visible light absorption by phytoplankton communities.

Over the past decades, an increase in the productivity and occurrence of potentially toxic cyanobacterial blooms has been observed in coastal and open waters of the Baltic Sea and some Polish Pomeranian lakes (e.g., Pliński et al. 1998). Massive cyanobacteria blooms occur almost every summer covering the area reaching up to 100,000 km^2^ (e.g., Kahru et al. 1994). *Nodularia spumigena*, *Aphanizomenon flos-aque* as well as some species of the genus *Anabaena* are the dominant cyanobacteria occurring in the Gulf of Gdańsk (Pliński et al. 2007), whereas *Microcystis* spp. are by far the most commonly occurring cyanobacteria in freshwater reservoirs (Kobos et al. 2005, 2013). Picocyanobacteria like *Synechococcus* spp. or *Synechocystis* spp. also play a significant role during blooms (Mazur-Marzec et al. 2013). Their biomass can pose up to 80 % of total bloom biomass, and they can be responsible for even 50 % of primary production in the bloom (e.g., Stal et al. 2003). Besides these dominating species, other species like *Pseudanabaena* spp. are also present in the Baltic Sea and freshwater reservoirs of cyanobacterial communities (Stal et al. 2003; Mazur-Marzec et al. 2013). Considering the production of biologically active compounds, both toxins and potential pharmaceuticals, the Baltic cyanobacteria have been intensively studied recently. Among the species considered in this study, cyanobacteria strain *N. spumigena* CCNP1401 produces the most active compounds, including proteases and phosphatase inhibitors (Mazur-Marzec et al. 2015). The extracts from the strains studied here usually cause protein phosphatase 1 inhibition (*Microcystis aeruginosa* CCNP 1101, *Anabaena* sp. CCNP 1406, *Synechocystis salina* CCNP 1104, *N. spumigena* CCNP 1401, *Nostoc* sp. CCNP 1411). Some of the strains produce protein phosphatase 2A, trypsin, thrombin, and carboxypeptidase-A inhibitors. The cyanobacterial compounds also have a strong impact on the growth of bacteria and fungi (Mazur-Marzec et al. 2015). It has been suggested that some of the Baltic alleged to be non-toxic. *Aphanizomenon flos-aquae* strains can produce bioactive compounds that can influence plankton composition and activity (Sellner 1997). A summary of bioactivity of studied cyanobacteria is given in Table 1.

**Table 1:** Metabolites detected (+) in the studied strains of cyanobacteria and the inhibition of enzymes by cyanobacterial extracts (for details, see Mazur-Marzec et al. 2015)

Strain	Species	MC	NOD	AP	AER	SPU	CPL	NCP	Inhibition
KAC 15	*A. flos-aquae*	Not analyzed							
CCNP 1101	*M. aeruginosa*	+			+				+
CCNP 1406	*Anabaena* sp.								+
CCNP 1104	*S. salina*								+
CCNP 1317	*Phormidium* sp.								
CCNP 1401	*N. spumigena*		+	+	+	+			+
CCNP 1108	*Synechococcus* sp.								+
CCNP 1411	*Nostoc* sp.						+	+	+
CCNP 1105	*Cyanobacterium* sp.								
CCNP 1312	*P. cf. galeata*								

*MC* microcystin, *NOD* nodularin, *AP* anabaenopeptin, *AER* aeruginosin, *SPU* spumigin, *CPL* cyanopeptolin, *NCP* nostocyclopeptide

Considering the high importance of the light absorption by phytoplankton from the point of view of productivity of water reservoirs, it is necessary to know the phytoplankton optical properties. The absorption coefficient of phytoplankton is somewhat difficult to determine, and therefore, many different techniques have been developed. The most common one is to collect phytoplankton cells on a glass fiber filter and determine the optical density of the collected material with the use of a spectrophotometer equipped with an integrating sphere (Tassan and Ferrari 1995; Röttgers and Gehnke 2012) and then to remove the pigments by extracting (Kishino et al. 1985) or oxidizing them (Tassan and Ferrari 1995). The phytoplankton absorption is obtained by subtracting the absorption by the bleached material from the absorption by total particulate matter. Another approach is to reconstruct the spectra using the information on the concentration of five main pigment groups, i.e., chlorophyll *a*, *b*, and *c* and photosynthetic and photoprotective carotenoids (Bidigare et al. 1990; Hoepffner and Sathyendranath 1991; Ficek et al. 2004). However, this approach does not account for the group of phycobilin pigments that are always present in cyanobacteria cells. Moreover, these water-soluble pigments are also present in different algal groups like red algae, cryptomonads, prochlorophytes, or glaucocytophytes (Roy et al. 2011). Considering the fact that the magnitude and the spectral shape of the absorption coefficient of phytoplankton result mostly from their photosynthetic and photoprotective pigment composition which is somewhat unique for certain phytoplankton groups which contain characteristic markers and accessory pigments (Mackey et al. 1996). Therefore, in recent years, a number of studies applying the statistical analysis of in vivo phytoplankton absorption spectra in taxonomical identification of water samples have been performed (Moberg et al. 2002; Lokuhewage et al. 2005; Lokuhewage and Fujino 2011). Their results showed that using the absorptive properties together with appropriate statistical approach can be a valuable tool in detecting phytoplankton groups and even species.

The aim of this study was to study and analyze the variability of the optical properties of cyanobacteria commonly occurring in Baltic Sea waters and to link these optical features with the pigment composition of studied cyanobacteria. Our study focused on the natural variability of these characteristics among phytoplankton from one taxonomical group grown under the same light and nutrient conditions. Our aim here was not to find the bio-optical method for distinguishing between selected cyanobacteria species and toxic and non-toxic species. The knowledge of such differences can be very useful in taxonomical identification of species and can be applied in creating algorithms for remote sensing techniques in monitoring of aquatic environment.

## Methods

### Culturing procedures

Ten strains of cyanobacteria (*A. flos-aquae* KAC 15, *M. aeruginosa* CCNP 1101, *Anabaena* sp. CCNP 1406, *S. salina* CCNP 1104, *Phormidium* sp. CCNP 1317, *N. spumigena* CCNP 1401, *Synechococcus* sp. CCNP 1108, *Nostoc* sp. CCNP 1411, *Cyanobacterium* sp. CCNP 1105, *Pseudanabaena cf. galeata* CCNP 1312) were analyzed. All analyzed strains, besides *A. flos-aquae*, came from the Culture Collection of Northern Poland of the Laboratory of Biochemical Ecology of Microorganisms from the University of Gdansk. The monoculture of *A. flos-aquae* KAC 15 was obtained from the University of Kalmar. All monocultures were grown under low light conditions (7 μmol photons m^−2^ s^−1^) with the use of Sylvania Luxline Plus Cool White de Luxe at the temperature of 22 ± 0.1 °C. Such low light intensity is comparable to that observed at the depth of ca. 5 m in Baltic waters when concentrations of suspended matter is high, e.g., during summer blooms. All analyzed organisms were grown in batch cultures in Z8 medium (Kotai 1972). In the case of *N. spumigena*, the medium was enriched with NaCl to obtain the salinity of 7 PSU. Before the measurements, the cultures were diluted daily for several days with fresh medium in order to maintain the cells in the exponential phase of growth and to minimize concentrations of detrital material. Moreover, the cell densities were adjusted to keep the cultures optically thin, in order to minimize shading and multiple scattering during absorption measurements.

### Optical measurements and spectra analysis

#### Suspension measurements

Optical measurements of the spectral absorption, *a*_*p*_(λ), and beam attenuation coefficient, *c*_*p*_(λ), were performed within the spectral region from 350 to 800 nm at 1-nm intervals with the use of Perkin Elmer Lambda 850 dual-beam spectrophotometer equipped with a 15-cm integrating sphere (Labsphere). The beam attenuation was measured with the cuvette placed about 20 cm from the integrating sphere entrance, and the geometry of measurement minimized the angle of acceptance for detecting light to less than 1° (e.g., Stramski and Piskozub 2003). The absorption measurements were made on samples of particle suspension in a 1-cm cuvette that was placed inside the integrating sphere (e.g., Stramski et al. 2002). Baseline spectra for both measurements were determined on the sample filtrate collected upon filtration through a prewashed 0.2-μm polycarbonate membrane filter (Nuclepore) in order to eliminate the influence of CDOM on the obtained spectra. All optical measurements were made on samples with optical density *OD* at 400 nm less than 0.3 in the beam attenuation setup to ensure the insignificant multiple scattering effects within the 1-cm cuvette (van de Hulst 1957). For each sample, scans of the optical density were made on at least three aliquots of particle suspension and the final sample spectrum was obtained by averaging the replicate scans. The particle spectral absorption coefficient *a*_*p*_(λ) (m^−1^) and the beam attenuation coefficient *a*_*p*_(λ) (m^−1^) were calculated by multiplying the appropriate baseline-corrected optical density values of the sample by 2.3 and dividing by the optical path length (0.01 m). The scattering coefficient of particles *b*_*p*_(λ) (m^−1^) was calculated as the difference between the beam attenuation coefficient and absorption coefficient. Specific absorption and scattering and beam attenuation coefficients were obtained by dividing the appropriate spectra by the chlorophyll *a* concentrations measured using the high-performance liquid chromatography (HPLC) as described in the following section.

Even though the cultures were grown under favorable conditions without nutrient limitation, some influence of detrital matter on the magnitude and the spectral shape of total absorption *a*_*p*_(λ) (m^−1^) was noted. The phytoplankton absorption was measured in the same way as the total particulate absorption after depigmentation of a sample by adding a solution of sodium hypochlorite as an oxidizing agent (e.g., Tassan and Ferrari 1995) against the sample filtrate after sample filtration through a prewashed 0.2-μm polycarbonate membrane filter as a blank. No decrease in the absorption of the filtrate was noted during filtration as observed in some studies (Röttgers et al. 2007); therefore, it could be used as a reference. In such a way, after multiplying the obtained optical densities by 2.3 and dividing by the cuvette length, the absorption spectra of detrital matter *a*_*det*_(λ) (m^−1^) were obtained. The phytoplankton absorption coefficient *a*_*ph*_(λ) (m^−1^) was obtained by subtracting the detrital absorption from the total absorption coefficient *a*_*p*_(λ) (m^−1^). After calculating the magnitudes of all coefficients, the spectra were smoothed with the use of Savitzky-Golay smoother with 11-nm window with the use of OriginPro software.

#### Filter technique

In the case of each cyanobacteria culture, the absorption spectra were measured also after collecting them onto a glass fiber filter. We measured both fresh and frozen cultures in order to check if this common method of sample preserving in liquid nitrogen or the absorption measurement procedure can affect the absorption spectra. The optical density in this case was measured after inserting the sample into the integrating sphere with use of a special commercially available clip style holder (e.g., Röttgers and Gehnke 2012). After the first measurement, the pigments were oxidized with the use of sodium hypochlorite, the oxidizing agent was washed out from the filter, and the optical density was measured again (e.g., Tassan and Ferrari 1995). The same procedure was performed for samples frozen in liquid nitrogen. This dataset was used to evaluate the measurement error connected to freezing and thawing samples during the measurement. The optical densities obtained for fresh (*OD*_fresh_) and frozen (*OD*_frozen_) samples were compared.

### Methodology of pigment analysis

Due to different physicochemical properties of tested pigment compounds, the appropriate methodology was used for their isolation, identification, and precise quantification in examined algae strains.

#### Extraction of pigments from phytoplankton cells

Two types of extraction media were used to isolate pigments from algal cells: 90 % acetone solution with respect to chlorophylls and carotenoids (Parsons et al. 1984) and extraction medium consisted of 0.25 M Trizma Base, hydrated 10 mM disodium EDTA (2H_2_O), and 2 mg cm^−3^ lysozyme; the initial pH 9 was adjusted to final 5.5 (HCl)—in case of extraction phycobiliproteins from cyanobacteria cells (according to Steward and Farmer 1984). Chlorophylls and carotenoids were extracted by mechanical grinding and sonication (2 min, 20 kHz, Cole Parmer, 4710 Series) in the darkness conditions at 4 °C for 2 h. Procedure of isolation of phycobiliproteins from cells was based on combination of a gentle mechanical grinding and enzymatic (lysozyme) reaction in order to successfully disintegrate cell walls and improve pigment extraction efficiency in darkened room conditions. Filers were then incubated at 37 °C for 2 h in a dry block heat bath (Thermoleader, Uniequip) and after that kept in dark at 4 °C for 24 h. The extract was then centrifuged (20 min, 5 °C, 3210*×g*, Beckman, GS-6R) to remove the filters and cellular debris.

The clarified extracts were then subjected to the chromatographic analysis in case of chlorophylls and carotenoids and spectrofluorometric measurements in case of phycobilins.

#### Qualitative and quantitative designation of chlorophylls and carotenoids in cyanobacteria cells

The chromatographic system HP1200 (Agilent, Perlan Technologies) used for chlorophyll and carotenoid isolation and separation was equipped with C_18_ LichroCARTLiChrospher100 RP18e (Merck) analytical column (dimension 250 × 4 mm, particle size 5 μm, and pore size 100 Å).

Pigments were isolated from extract by use of reverse-phase high-performance liquid chromatography (RP-HPLC), commonly used to analyze samples with wide polarity components, such pigments are. The method of pigment isolation and separation was introduced by Mantoura and co-workers (Mantoura and Llewellyn 1983), adopted and modified in later years by other researchers (Barlow et al. 1993; Stoń and Kosakowska 2002; Stoń-Egiert and Kosakowska 2005). Calibration of chromatographic system was based on commercially available chlorophylls and carotenoids (The International Agency for ^14^C Determination DHI Institute for Water and Environment in Denmark).

#### Qualitative and quantitative designation of phycobiliproteins in cyanobacteria cells

Concentrations of phycocyanin and phycoerythrin were determined on the basis of spectrofluorometric measurements (Cary Eclipse, Varian, Agilent Technologies) of previously prepared extracts, which allows for obtaining spectra of fluorescence emission (500–700 nm) with resolution of 2 nm were obtained every 5 nm of excitation.

The calibration parameters and response factors for appropriate wavelengths of extinction and emission curves were performed for commercially available phycobilin standards (ProZyme Inc., USA) prepared in medium solution. The calibration analysis was carried out for three settings of excitation signal controlled by varying intense of xenon lamp, set at 600, 800, and 1000 V. A detailed description of the methodology used is given in the work by Sobiechowska-Sasim et al. (2014).

### Size distribution determination

Concentration of particles in known volume of analyzed samples, as well as the volumetric concentration of cyanobacteria cells, was obtained by the use of Multisizer 4 Coulter Counter (Beckman Coulter) equipped with a 100-μm aperture (particle size range from 2 to 60 μm). The device uses the Coulter method, known as electrical sensing zone (ESZ), a method with high resolution and accuracy, additionally supported by digital pulse processor, which provides ultra-resolution, multi-channel analysis, and accuracy unattainable by other technologies and methods of measurement (volumetric pump precise is higher than 99.5 %). The application of 100 μm aperture allows for precise designation of particles in 2–60 μm size range (treated as equivalent sphere diameter) divided into 400 size channels logarithmically spaced over the measured range. The aperture was calibrated by the use of internal standard, latex beads with modal size 19.66 μm (Beckman Coulter), prepared in recommended concentration in double filtered through membrane filters (pore size 0.2 μm) of artificial seawater (salinity 7 PSU). The measurements of particle size distribution (PSD) were made in three replicates for each sample using volumetric control mode of measurements (from 0.05 to 2 cm^3^ depending on particle content).

## Results

### Optical properties of cyanobacteria strains

**Fig. 1:**
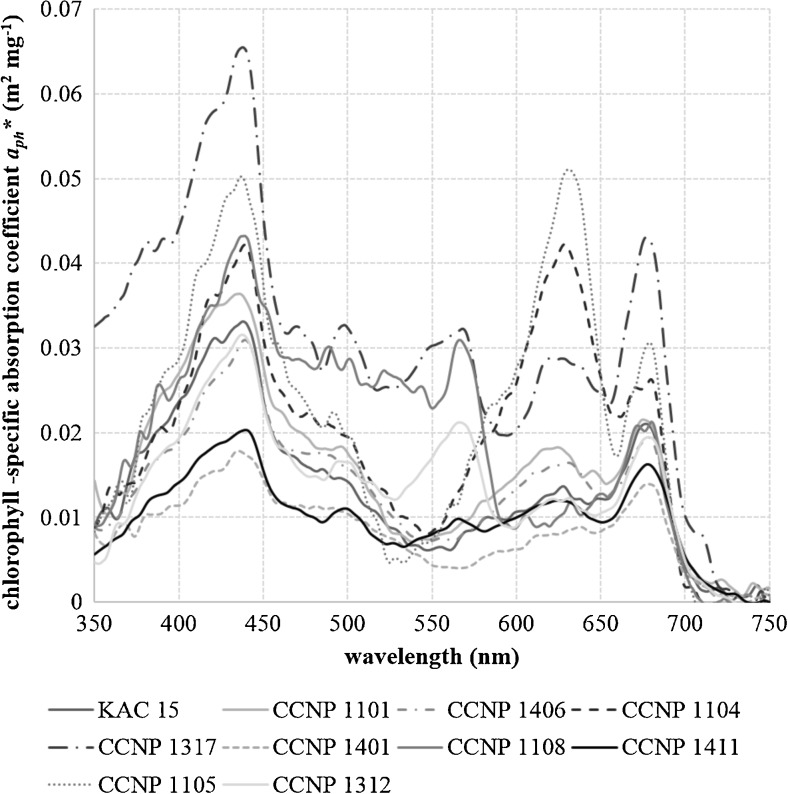
Chlorophyll-specific absorption spectra of cyanobacteria strains: *Aphanizomenon flos-aquae* KAC 15, *Microcystis aeruginosa* CCNP 1101, *Anabaena* sp. CCNP 1406, *Synechocystis salina* CCNP 1104, *Phormidium* sp. CCNP 1317, *Nodularia spumigena* CCNP 1401, *Synechococcus* sp. CCNP 1108, *Nostoc* sp. CCNP 1411, *Cyanobacterium* sp. CCNP 1105, *Pseudanabaena cf. galeata* CCNP 1312

The absorption features of the analyzed cyanobacteria strains differed both in the magnitude and the spectral shape of the absorption coefficient (Fig. 1, Table 2). On the average, the highest chlorophyll-specific absorption coefficient was observed in the case of *Phormidium* sp. CCNP 1317 (0.065 m^2^ mg^−1^), whereas the lowest for *N. spumigena* CCNP 1401 (0.017 m^2^ mg^−1^) and *Nostoc* sp. CCNP 1411 (0.020 m^2^ mg^−1^).

**Table 2:** Basic absorptive properties of ten cyanobacteria strains at 440, 630, and 675 nm

Strain	Species	*a*_ph_*(440)	*a*_ph_*(630)	*a*_ph_*(675)	*a*_*ph*_(440): *a*_*ph*_(675)
		(m^2^ mg^−1^)	(m^2^ mg^−1^)	(m^2^ mg^−1^)	–
KAC 15	*A. flos-aquae*	0.033	0.013	0.021	1.567
CCNP 1101	*M. aeruginosa*	0.036	0.018	0.022	1.658
CCNP 1406	*Anabaena* sp.	0.031	0.017	0.019	1.623
CCNP 1104	*S. salina*	0.042	0.042	0.025	1.691
CCNP 1317	*Phormidium* sp.	0.065	0.028	0.042	1.530
CCNP 1401	*N. spumigena*	0.017	0.008	0.014	1.253
CCNP 1108	*Synechococcus* sp.	0.043	0.011	0.020	2.135
CCNP 1411	*Nostoc* sp.	0.020	0.012	0.016	1.271
CCNP 1105	*Cyanobacterium* sp.	0.048	0.051	0.030	1.640
CCNP 1312	*P. cf. galeata*	0.031	0.012	0.019	1.617

The magnitude of *a*_ph_*(675) peak is mainly attributed to the chlorophyll *a* and the package effect; however, in the case of cyanobacteria, it can be slightly affected by phycocyanin, whereas the *a*_ph_*(440) peak is influenced additionally by the accessory pigments. Therefore, the variations in its magnitude result from the pigment composition and content in a phytoplankton cell as well as from the package effect (e.g., Bricaud et al. 1995). The lowest values of *a*_ph_*(675) were observed for large-celled filamentous cyanobacterium *N. spumigena* (Table 1) which because of the size of the cells is mostly affected by the package effect. According to Bricaud et al. (1995), the dimensionless factor describing the package effect *Q*_a_*(675) was calculated. The lower the value of this factor, the higher is the influence of the package effect on the absorption coefficient spectra. For *N. spumigena*, the value of *Q*_a_*(675) was the lowest and equaled 0.67. In other cases, the value of *Q*_a_*(675) was close to 1 which means that the package effect has low influence on the absorption properties of studied species. In the case of *Phormidium* sp., the value was 2.05 which is impossible because the limiting value of this coefficient is 1. However, such values were also observed and could be caused by the absorption measurement technique inaccuracy or the influence of other pigments (Bricaud et al. 1995). The blue to red ratio *a*_*ph*_(440) : *a*_*ph*_(675) that can be treated as the factor describing the relative (to chlorophyll *a*) contribution of accessory pigments to total absorption ranged between 1.25 (*N. spumigena*) to 2.14 (*Synechococcus* sp.) (Table 2).

Some of the analyzed strains were characterized by considerably high absorption coefficient values at 630 nm, especially *Cyanobacterium* sp. CCNP 1105 and *S. salina* CCNP 1104 whose absorption coefficient values were as high as 0.051 and 0.042 m^2^ mg^−1^, respectively (Table 1). In the case of *Cyanobacterium* sp., the 630-nm peak was higher than both chlorophyll *a* absorption peaks (Fig. 1, Table 2).

**Fig. 2:**
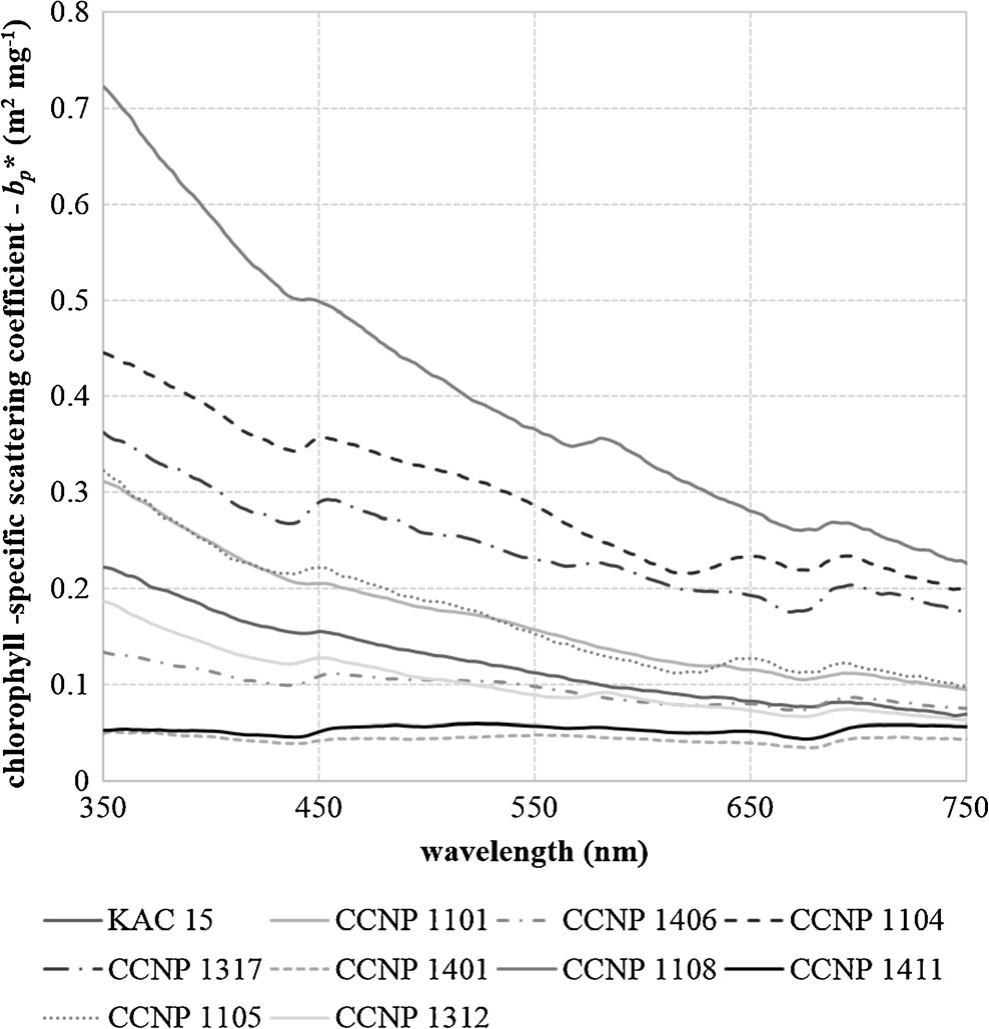
Chlorophyll-specific scattering spectra of cyanobacteria strains: *Aphanizomenon flos-aquae* KAC 15, *Microcystis aeruginosa* CCNP 1101, *Anabaena* sp. CCNP 1406, *Synechocystis salina* CCNP 1104, *Phormidium* sp. CCNP 1317, *Nodularia spumigena* CCNP 1401, *Synechococcus* sp. CCNP 1108, *Nostoc* sp. CCNP 1411, *Cyanobacterium* sp. CCNP 1105, *Pseudanabaena cf. galeata* CCNP 1312

The analyzed cyanobacteria differed in the ability to scatter the light as well (Fig. 2). The highest and the most steep scattering coefficient spectrum was observed for the smallest, single-celled species *Synechococcus* sp. and *S. salina* (Fig. 2, Table 3). In the case of both these species, the average contribution of the absorption into total light attenuation was lower than 9 %. The most flat spectra with almost no light wavelength dependency were noted for *N. spumigena* and *Nostoc* sp. (Fig. 2). These organisms were also characterized by the largest sizes of their filaments (see Fig. 6).

**Table 3:** Selected light scattering and attenuation properties by ten cyanobacteria strains

Strain	Species	*b*_p_*(600) (m^−1^)	*a*_*p*_(λ)/*c*_*p*_(λ)		
			Average	Min	Max
KAC 15	*Aphanizomenon flos-aquae*	0.093	0.118	0.030	0.235
CCNP 1101	*Microcystis aeruginosa*	0.129	0.100	0.032	0.182
CCNP 1406	*Anabaena* sp.	0.082	0.132	0.015	0.262
CCNP 1104	*Synechocystis salina*	0.231	0.088	0.013	0.179
CCNP 1317	*Phormidium* sp.	0.212	0.115	0.008	0.218
CCNP 1401	*Nodularia spumigena*	0.044	0.187	0.019	0.363
CCNP 1108	*Synechococcus* sp.	0.333	0.067	0.017	0.107
CCNP 1411	*Nostoc* sp.	0.053	0.166	0.008	0.336
CCNP 1105	*Cyanobacterium* sp.	0.119	0.145	0.025	0.336
CCNP 1312	*Pseudanabaena cf. galeata*	0.083	0.131	0.008	0.238

In order to compare the magnitude and spectral shape of the absorption coefficient of analyzed cyanobacteria strains, we used the similarity index (SI) defined as follows (Millie et al. 2002):

1 $$ \mathrm{S}\mathrm{I}\kern0.5em =\kern0.5em 1-\frac{2}{\pi}\mathrm{arcos}\;\frac{{\displaystyle \sum {a}_i{b}_i}}{\sqrt{{\displaystyle \sum {a}_i^2}\kern0.5em {\displaystyle \sum {b}_i^2}}} $$

where *a*_*i*_ and *b*_*i*_ are the absorption coefficients at *λ*_*i*_*.*

Despite the fact that all analyzed strains were cyanobacteria, a considerable variability in the spectral shape and magnitude of absorption coefficient was noted (Fig. 1, Table 4).

**Table 4:** Similarity between analyzed cyanobacteria spectra within the 350–750 nm band

	KAC 15	CCNP 1101	CCNP 1406	CCNP 1104	CCNP 1317	CCNP 1401	CCNP 1108	CCNP 1411	CCNP 1105	CCNP 1312
KAC 15	1	0.94	0.91	0.78	0.87	0.90	0.77	0.88	0.77	0.83
CCNP 1101	–	1	0.93	0.81	0.87	0.89	0.76	0.89	0.80	0.83
CCNP 1406	–	–	1	0.83	0.87	0.93	0.78	0.91	0.81	0.84
CCNP 1104	–	–	–	1	0.76	0.79	0.67	0.82	0.92	0.74
CCNP 1317	–	–	–	–	1	0.87	0.82	0.89	0.74	0.88
CCNP 1401	–	–	–	–	–	1	0.78	0.89	0.76	0.83
CCNP 1108	–	–	–	–	–	–	1	0.77	0.64	0.88
CCNP 1411	–	–	–	–	–	–	–	1	0.80	0.87
CCNP 1105	–	–	–	–	–	–	–	–	1	0.72
CCNP 1312	–	–	–	–	–	–	–	–	–	1

The most similar absorption spectra were observed in the case of *A. flos-aquae* and *M. aeruginosa* (SI = 0.94), whereas the most different spectra were noted for *S. salina* and *Synechococcus* sp. (SI = 0.67). Overall, the most different among all analyzed strains were the spectra of absorption by *Phormidium* sp. and *P. cf. galeata*, which can be also clearly seen in Fig. 1.

### Pigment composition in cyanobacteria

**Fig. 3:**
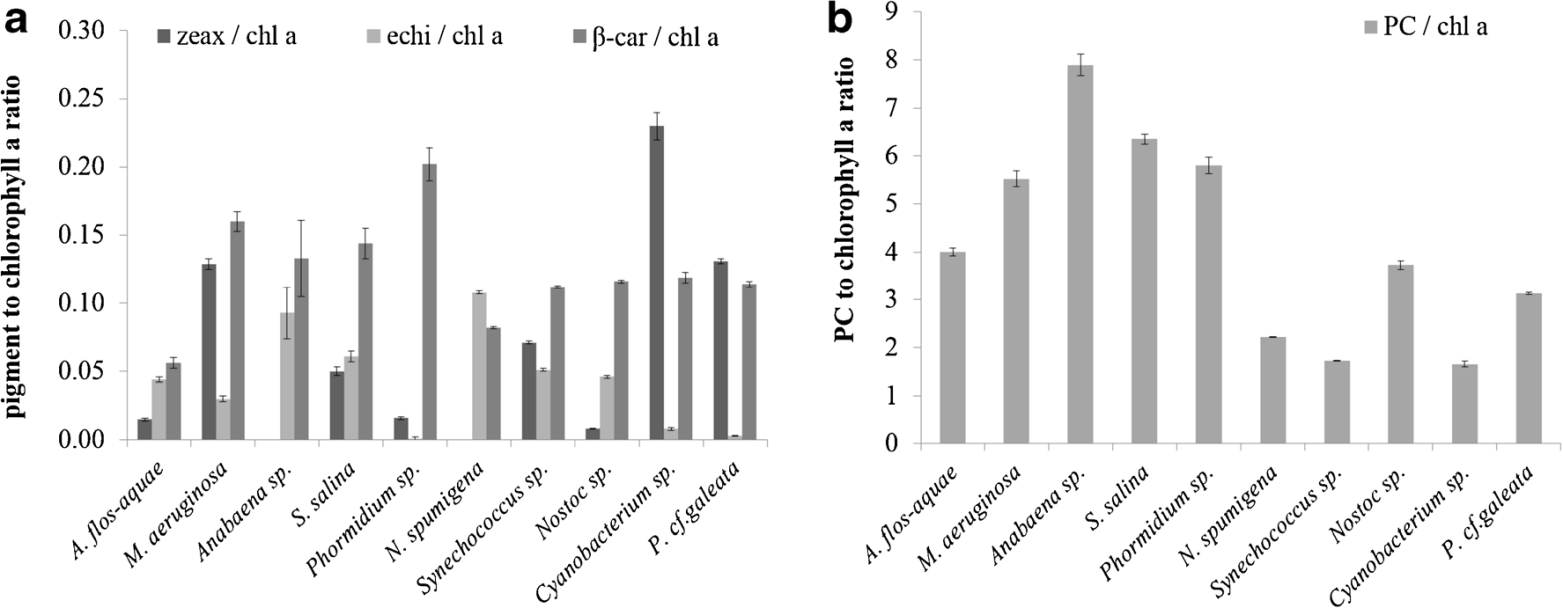
Ratios of the zeaxanthin, echinenone, *β*-carotene, and phycocyanin concentration to chlorophyll *a* concentration (±standard deviation) in analyzed strains of cyanobacteria

The concentration of chlorophyll *a*, carotenoids, and phycobilins was measured separately for every analyzed strain. On the average, 88.55 % of detected pigments were identified. Chlorophyll *a* concentration varied from 47.2 (*Cyanobacterium* sp.) to 246.5 mg m^−3^ (*Nostoc* sp.). Lower values of concentration of this pigment were usually observed for small-celled strains. Besides chlorophyll *a*, only echinenone, *β*-carotene, and phycocyanin were present in all analyzed strains (Table 5). The concentration of phycocyanin was much higher than concentrations of other pigments, e.g., for *Anabaena* sp., it was almost eight times higher than the chlorophyll *a* concentration. Zeaxanthin, which is widely used as a marker pigment for cyanobacteria (e.g., Schagerl and Müller 2006), was observed in eight out of ten analyzed strains with the maximum concentration of 18.3 mg m^−3^ observed in the case of *P. cf. galeata.* It was absent in *N. spumigena* and *Anabaena* sp., which are the species that occur in the Baltic Sea most frequently. Small amounts of zeaxanthin were also observed in previous studies on pigment production in blue-green algae (Goodwin 1957). Together with large ranges of pigment concentrations observed for various strains (Fig. 3), it could be observed that also the ratio of accessory pigments to chlorophyll *a* was not constant, especially in the case of *β*-carotene and echinenone. This can indicate different mechanisms of photoacclimation in the analyzed strains.

**Table 5:** Pigment composition (±standard deviation) in the analyzed cyanobacteria strains

Strain	Species	Chlide	Allox	Zeax	Canthax	Chl a	Echi	β-car	PE	PC
		(mg m^−3^)	(mg m^−3^)	(mg m^−3^)	(mg m^−3^)	(mg m^−3^)	(mg m^−3^)	(mg m^−3^)	(mg m^−3^)	(mg m^−3^)
KAC 15	*A. flos-aquae*			1.26 (±0.24)	1.09 (±0.24)	83.85 (±12.39)	3.70 (±0.52)	4.70 (±0.67)		329.57 (±29.60)
CCNP 1101	*M. aeruginosa*			12.46 (±1.91)		96.14 (±13.09)	2.85 (±0.51)	15.27 (±1.45)		517.47 (±98.61)
CCNP 1406	*Anabaena* sp.				0.73 (±0.11)	137.83 (±35.17)	12.48 (±1.83)	17.73 (±2.81)		1038.01 (±43.01)
CCNP 1104	*S. salina*			3.08 (±0.32)	0.16 (±0.03)	61.73 (±8.62)	3.75 (±0.39)	8.82 (±0.88)		386.61 (±12.90)
CCNP 1317	*Phormidium* sp.			1.14 (±0.29)		72.14 (±16.24)	0.10 (±0.04)	14.66 (±3.86)	215.69 (±12.48)	400.07 (±18.23)
CCNP 1401	*N. spumigena*				3.40 (±0.12)	128.17 (±2.75)	13.87 (±0.23)	10.55 (±0.16)		284.23 (±18.67)
CCNP 1108	*Synechococcus* sp.			3.23 (±0.26)		45.86 (±3.62)	2.31 (±0.12)	5.13 (±0.37)	91.75 (±6.17)	79.02 (±4.70)
CCNP 1411	*Nostoc* sp.	1.00 (±0.31)	3.28 (±0.68)	1.88 (±0.31)		246.52 (±46.28)	11.33 (±1.96)	28.69 (±5.44)	239.59 (±10.82)	893.22 (±50.68)
CCNP 1105	*Cyanobacterium* sp.	1.01 (±0.68)	3.02 (±0.69)	10.87 (±2.09)		47.23 (±9.68)	0.38 (±0.07)	5.61 (±1.18)		74.30 (±13.56)
CCNP 1312	*P. cf. galeata*	1.21 (±0.50)	2.79 (±0.23)	18.34 (±1.51)		140.42 (±13.37)	0.40 (±0.05)	15.99 (±1.44)	454.03 (±17.00)	427.33 (±17.07)

All statistics obtained for *n* = 3 replicates

*Chlide* chlorophyllide a, *Allox* alloxanthin, *Zeax* zeaxanthin, *Canthax* canthaxanthin, *Chl a* chlorophyll a, *Echi* echinenone, *β-car β*-carotene, *PE* phycoerythrin, *PC* phycocyanin

**Fig. 4:**
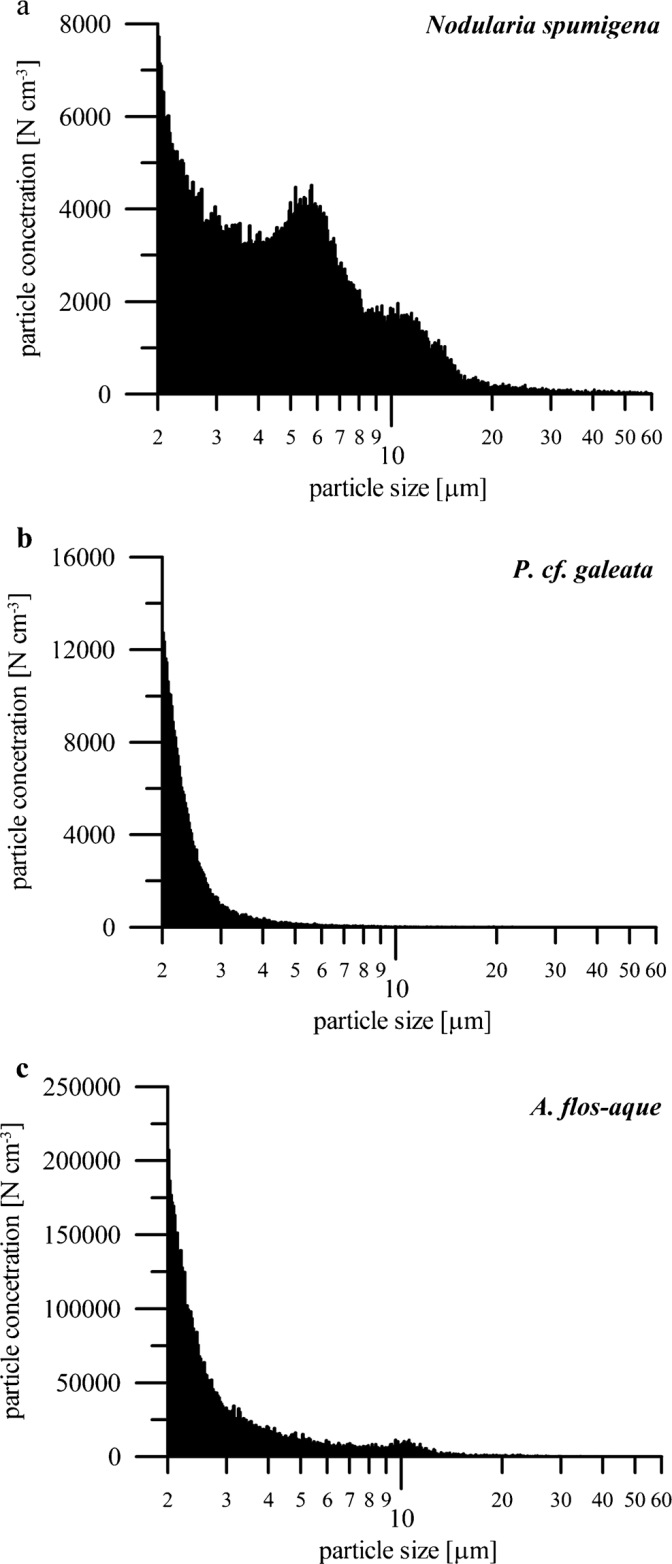
Size distributions of cells/filaments of selected cyanobacteria: **a** *Nodularia spumigena*, **b** *Pseudanabaena cf. galeata*, and **c** *Aphanizomenon flos-aquae*

Considering the number of particles (single cells or filaments) in analyzed samples within the 2–60 μm range, the concentrations of selected pigments were recalculated (Table 6). The number of detected particles within this size range varied between 1.3 × 10^5^ particles cm^-3^ (SD = 0.008 × 10^5^ particles cm^-3^) for *N. spumigena* to 77.2 × 10^5^ particles cm^-3^ (SD = 4.9 × 10^5^ particles cm^-3^) for *Nostoc* sp. The highest concentrations of all detected pigments in one filament were recorded for *N. spumigena* mainly because of the fact that this species was characterized by the largest filaments among all analyzed cyanobacteria (Fig. 4a). However, it is worth to notice that similarly high values of pigments in one filament were observed for much smaller *P. cf. galeata* (Fig. 4b). The lowest concentrations of all detected pigments were observed for *A. flos-aquae* characterized by rather large sizes (Fig. 4c). Considering these observations, we could not find any dependency between the size of cells/filaments and pigment concentration. However, it is necessary to emphasize here that the results from the Coulter counter should be only treated as an approximation, especially in the case of filamentous species, because of the measurement technique constraints.

**Table 6:** Concentration of selected pigments in single cell or filament of analyzed cyanobacteria strains

Strain	Species	Chlide	Allox	Zeax	Canthax	Chl a	Echi	β-car
		(mg per cell/fil) × 10^12^	(mg per cell/fil.) × 10^12^	(mg per cell/fil.) × 10^12^	(mg per cell/fil.) × 10^12^	(mg per cell/fil.) × 10^12^	(mg per cell/fil.) × 10^12^	(mg per cell/fil.) × 10^12^
KAC 15	*A. flos-aquae*			0.19	0.16	12.40	0.55	0.70
CCNP 1101	*M. aeruginosa*			22.38		172.70	5.12	27.43
CCNP 1406	*Anabaena* sp.				0.21	39.28	3.56	5.05
CCNP 1104	*S. salina*			4.87	0.25	97.56	5.93	13.94
CCNP 1317	*Phormidium* sp.			3.73		236.13	0.33	47.99
CCNP 1401	*N. spumigena*				25.52	962.04	104.11	79.19
CCNP 1108	*Synechococcus* sp.			4.33		61.49	3.10	6.88
CCNP 1411	*Nostoc* sp.	0.13	0.42	0.24		31.93	1.47	3.72
CCNP 1105	*Cyanobacterium* sp.	0.49	1.46	5.26		22.85	0.18	2.71
CCNP 1312	*P. cf. galeata*	4.53	10.45	68.69		525.89	1.50	59.88

*Chlide* chlorophyllide a, *Allox* alloxanthin, *Zeax* zeaxanthin, *Canthax* canthaxanthin, *Chl a* chlorophyll a, *Echi* echinenone, *β-car β*-carotene

### Preservation influence on the spectral shape of phytoplankton absorption

**Fig. 5:**
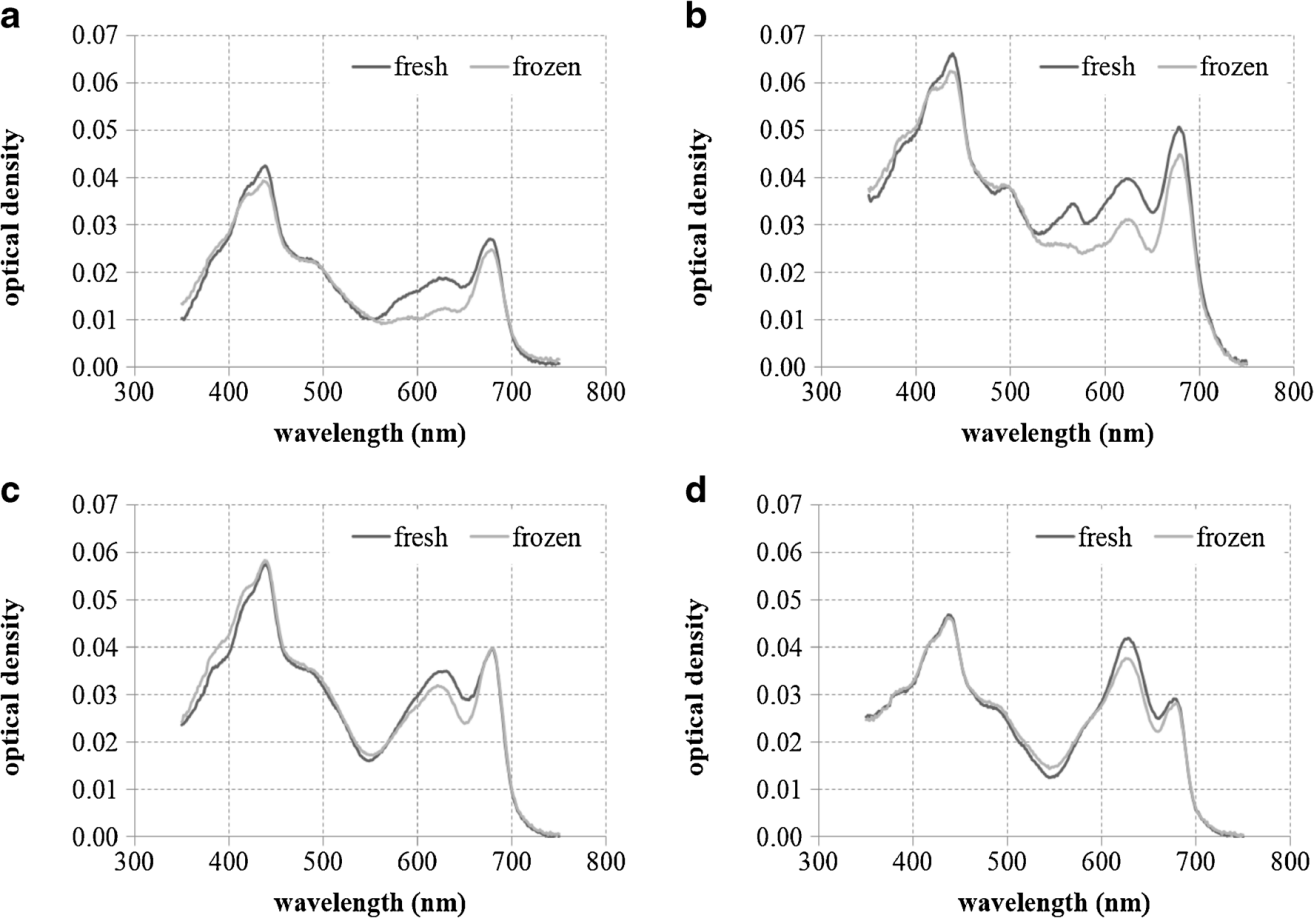
Comparison of the optical density spectra measured using the filter technique for fresh and frozen cyanobacteria monoculture samples: **a** *A. flos-aquae*, **b** *M. aeruginosa*, **c** *Anabaena* sp., and **d** *Nostoc* sp.

The comparison of the spectral shape and magnitude of the optical densities obtained for fresh and frozen in liquid nitrogen samples revealed some distinct differences in the case of some strains, as presented in Table 7 and Fig. 5. The lowest differences were observed for *Anabaena* sp., whereas the highest for *P. cf. galaeta*. For the latter strain, the differences were present within the entire spectrum similarly to the *N. spumigena* spectra. However, for the rest of the analyzed species, the differences around 630 nm were the highest, i.e., within the spectral region where the absorption by phycobilins are the strongest. However, when the concentrations of some pigments (both carotenoids and phyciobilins) were compared, there were no statistically significant differences among them (Table 8). We suspect that the error in this measurement method can be introduced during the thawing of the filters before the absorption measurement.

**Table 7:** Relative differences in the optical densities for measurements of absorption using the filter technique for fresh and frozen samples

Strain	Species	Relative difference		
		440 nm	630 nm	675 nm
KAC 15	*Aphanizomenon flos-aquae*	7.5 %	33.8 %	8.8 %
CCNP 1101	*Microcystis aeruginosa*	6.8 %	27.2 %	9.5 %
CCNP 1406	*Anabaena* sp.	−2.0 %	10.6 %	−0.9 %
CCNP 1104	*Synechocystis salina*	1.2 %	10.0 %	3.2 %
CCNP 1317	*Phormidium* sp.	3.3 %	16.5 %	6.3 %
CCNP 1401	*Nodularia spumigena*	16.6 %	19.8 %	15.5 %
CCNP 1411	*Nostoc* sp.	5.1 %	21.9 %	11.7 %
CCNP 1312	*Pseudanabaena cf. galeata*	25.6 %	37.9 %	26.2 %

**Table 8:** Results of the non-parametric Wilcoxon matched pairs test for the concentrations of chosen pigments in fresh and frozen cyanobacteria monoculture samples

Pigment	*n*	*Z*	*p*
Zeaxanthin	8	1.12	0.26
Chlorophyll *a*	10	1.17	0.24
Echinenone	10	0.66	0.51
*β*-carotene	10	0.15	0.88
PC	10	1.17	0.24
PE	4	1.46	0.14

The Wilcoxon test is significant at the 0.01 level

*n* number of samples, *Z* test statistic, *p* confidence level

## Discussion

The spectral shape and magnitude of the absorption coefficient reflects the phytoplankton pigment composition. Therefore, the absorption or its fourth spectra are often used in order to detect specific pigments and/or identify some phytoplankton species or groups (e.g., Aguirre-Gómez et al. 2001). In the case of phytoplankton containing many different pigments with overlapping absorption bands, the use of derivative analysis in order to determine quantitatively the position and the intensity of the absorption peaks is the easiest and the most convenient way. It is worth to mention that the absorption peaks of particular pigments measured in vivo and in solution are usually shifted, usually by about 10 nm. However, the difference in the location of absorption peaks can even reach 50 nm (Bidigare et al. 1990; Smith and Alberte 1994). This effect can be caused by thylakoid membrane degradation as well as disintegration of pigment-protein complexes during pigment extraction (Markwell and Thornber 1982; Smith and Alberte 1994). The magnitude of this shift depends on the type of solvent (e.g., Woźniak and Dera 2007) and the degree of protein complexe degradation (e.g., Markwell and Thornber 1982). For example, in the case of a diatom *Phaeodactylum tricornutum*, the location of the chlorophyll *a* maximum in the long wave part of the spectrum varied between 667 and 677 nm depending on the type of extracted pigment-protein complex (Owens and Wold 1986). Therefore, it seems very important to focus on the in vivo pigment absorption, because it can be very useful in reconstructing phytoplankton absorption spectra based on the HPLC-derived pigment composition.

**Fig. 6:**
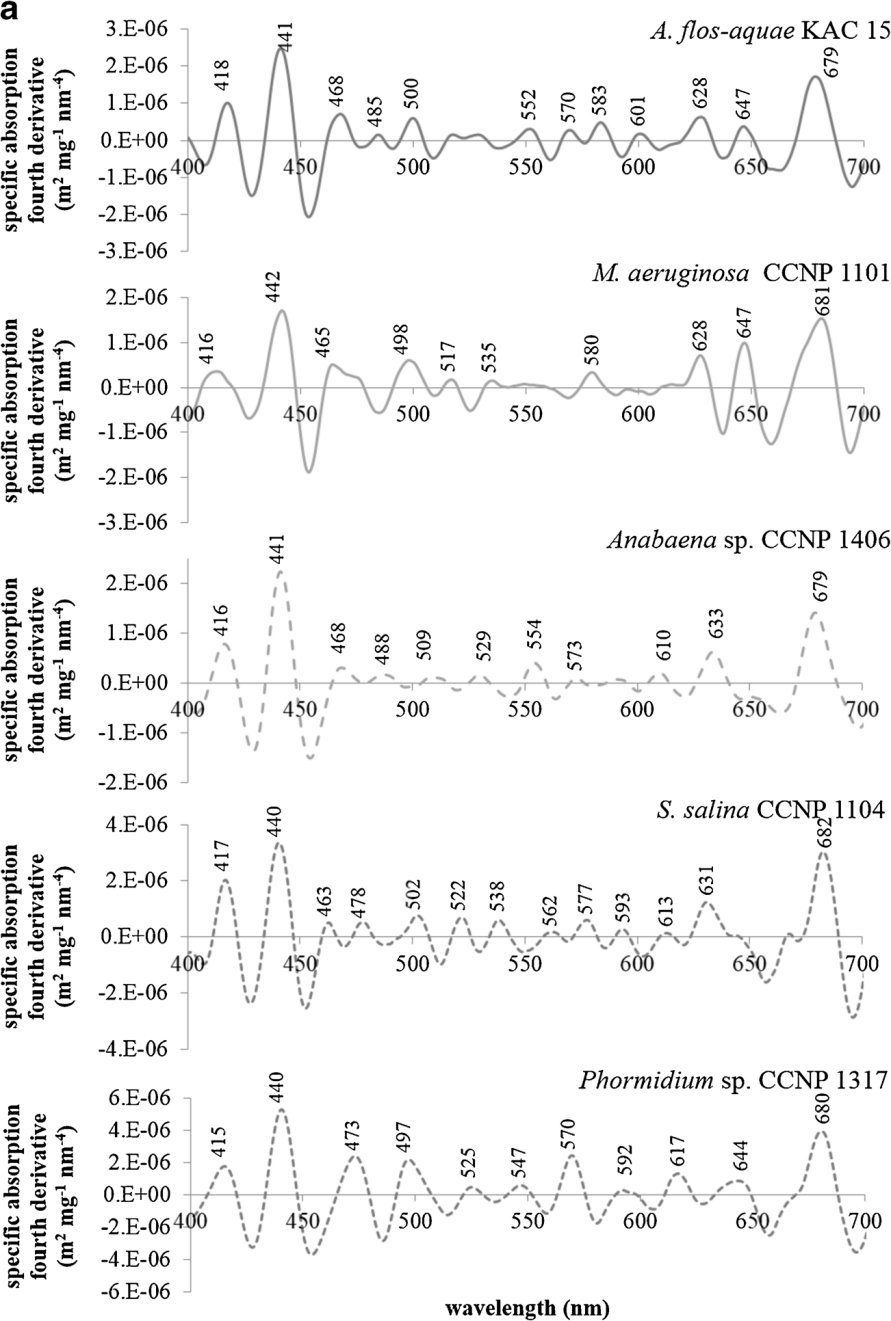

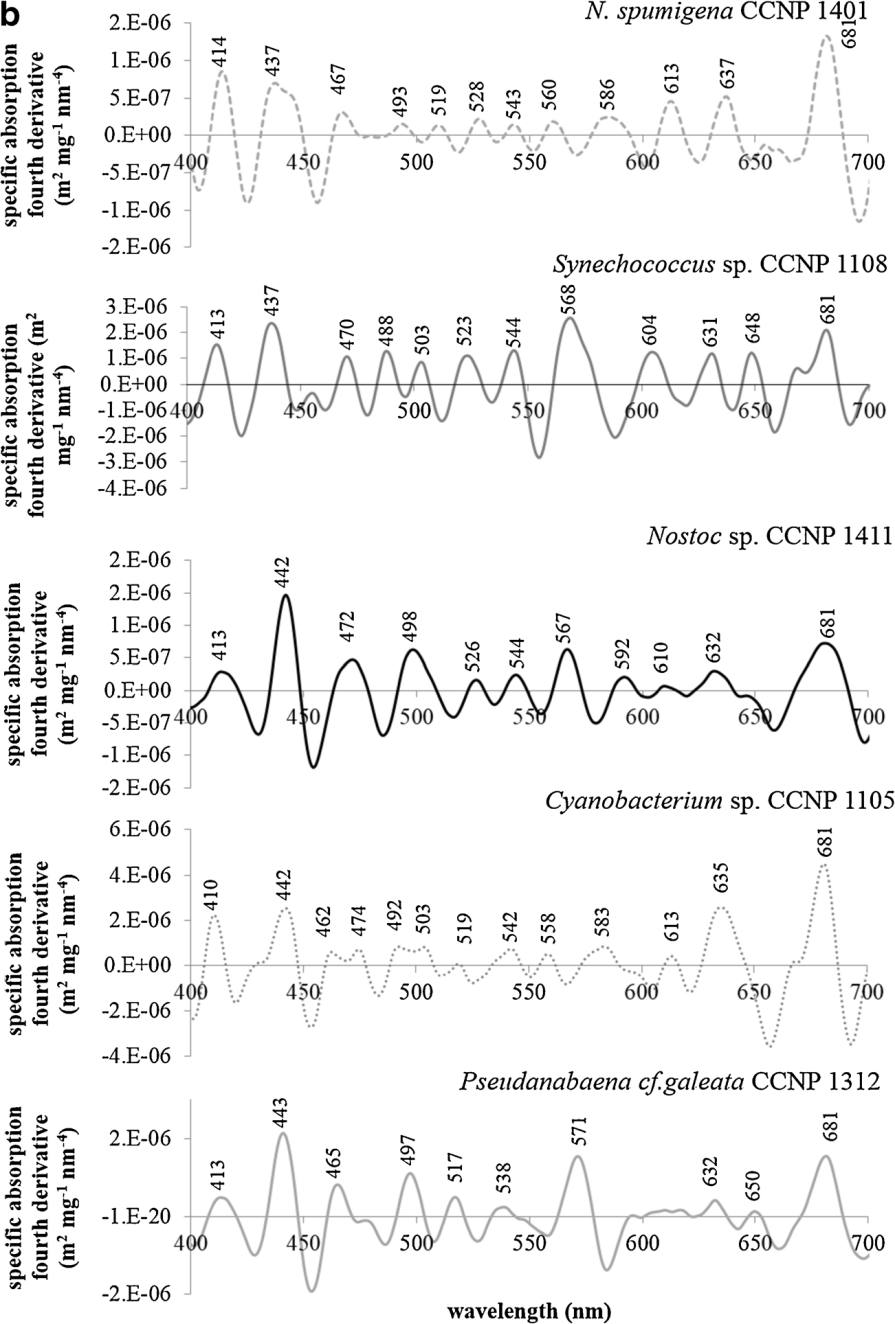
Chlorophyll-specific absorption fourth derivative spectra of cyanobacteria strains: *Aphanizomenon flos-aquae* KAC 15, *Microcystis aeruginosa* CCNP 1101, *Anabaena* sp. CCNP 1406, *Synechocystis salina* CCNP 1104, *Phormidium* sp. CCNP 1317, *Nodularia spumigena* CCNP 1401, *Synechococcus* sp. CCNP 1108, *Nostoc* sp. CCNP 1411, *Cyanobacterium* sp. CCNP 1105, *Pseudanabaena cf. galeata* CCNP 1312

The absorption of chlorophyll *a* has been studied for many years. In one of the first works, the absorption peaks were observed at 418, 437, 618, and 673 nm (Goedheer 1970). However, in more recent papers on various phytoplankton groups, the absorption maxima of this pigment were noted at 412, 435, 623, and 675 (Hoepffner and Sathyendranath 1991) or 415–425, 440–455, 620, and 675 nm (Aguirre-Gómez et al. 2001). In this study, we applied the fourth derivative approach to the phytoplankton absorption spectra and we observed distinct absorption peaks of chlorophyll *a* at 410–418, 437–443, and 679–681 nm (Fig. 6). The absorption maximum observed in previous studies at about 620 nm can be associated with the peaks between 610 and 632 nm. The main difference observed here is the shift in the long wave in vivo chlorophyll absorption maximum to about 680 nm. However, the differences in the location of this absorption peak among various groups of phytoplankton can be the result of proportion between photosystem I (PS I) and photosystem II (PS II) characterized by different absorptive capabilities (e.g., Hoepffner and Sathyendranath 1991). In all analyzed strains, absorption peaks at 462–474, 492–509, and 517–529 nm were observed. These absorption bands can be clearly associated with carotenoids and xanthophylls like *β*-carotene, zeaxanthin, alloxanthin, and aphanizophyll. Such observation was also made by Aguirre-Gómez et al. (2001) who assigned the in vivo absorption maxima around 490 and 536 nm to a mixture of pigments from the carotenoid and xanthophyll groups. According to Bidigare et al. (1990), the first absorption band is dominated by the absorption of light by *β*-carotene. Can be proven by the fact that in the case of *Phormidium* sp. for which the relative concentration of *β*-carotene in relation to chlorophyll *a* was the highest (Fig. 3) and the concentrations of remaining pigments were relatively low (Table 5), the fourth derivative peak at 473 nm is clearly visible (Fig. 6). The peak between 567 and 571 nm is clearly caused by the presence of phytoerythrin because it can be noticed only in the case of cyanobacteria for which this pigment was detected (Table 5). This information can be valuable for models for detecting this pigment; however, it does not occur in all cyanobacteria, so it will not be useful in algorithms for cyanobacteria biomass assessment. The last but very distinct absorption peak is located near 630 nm. This absorption band can be clearly associated with the presence of phycyanin (Fig. 6) whose in vivo absorption maximum has been observed at 612–626 nm (e.g., Simis and Kauko 2012).

The fourth derivative analysis of the absorption spectra has been also used in order to approximate the concentration of some pigments (Bidigare et al. 1989; Peréz et al. 2007). In our results, no significant correlation between the value of the fourth derivative and pigment concentrations were observed, even in the case of the chlorophyll *a* concentration for which such relations were noted in natural samples (Bidigare et al. 1989; Peréz et al. 2007). However, such result can be caused by very low amount of data, and it is necessary to analyze natural samples collected in the areas where cyanobacterial blooms occur.

The use of the fourth derivative analysis can be a valuable tool for determining the location of absorption peaks. However, as it could be observed in the analyzed absorption spectra, the bands of maximum absorption can change depending on the size and shape of organisms. Therefore, the spectra of phytoplankton absorption obtained through the reconstruction based on the HPLC-derived pigment concentration should be treated very carefully. It is very possible that the use of this analysis can be very useful in the case of detecting phytoplankton species in natural samples, especially those characterized by the presence of specific pigments in high concentrations. For example, the derivative analysis has been successfully used in detecting bloom-forming dinoflagellates like *Gymnodinium breve* or *Karenia mikimotoi* (Millie et al. 1997; Kirkpatrick et al. 2000; Staehr and Cullen 2003). For the time being, there have been no such trials performed for the Baltic Sea cyanobacteria species. However, such a laboratory approach as we presented here is very useful and should be treated as the first step in the analysis of natural samples which very often lack sharply defined peaks and shoulders which can be clearly seen in laboratory-prepared phytoplankton cultures (e.g., Bidigare et al. 1990).

The main constraint in applying the optical methods for phytoplankton species detection is the lack of knowledge of the variability in absorption and scattering properties under natural conditions. There have been several studies made on the laboratory-grown cyanobacteria cultures (Table 9); however, there is still not much consistency in the obtained results. The changes in the optical properties of phytoplankton cells induced by the light intensity and resulting mainly from changes in pigment composition are a very complicated problem. For some species, the magnitude of the chlorophyll-specific absorption coefficient is almost constant despite highly changing light conditions (e.g., Geider et al. 1985). Whereas for the other species, the observed changes can be very rapid. For example, a 4.5-fold increase in the *a*_p_*(440) value was observed in the case of *Synechocystis* sp. when the light intensity was changed from 20 to 700 μmol photons m^−2^ s^−1^ (Stramski and Morel 1990). It has been observed that the change in pigment composition can be responsible for 14–80 % variability in the magnitude of *a*_p_*(440) (Babin et al. 1996; Lazzara et al. 1996; Allali et al. 1997; Millán-Núñez et al. 1998; Stuart et al. 1998). Generally, phytoplankton grown under low light conditions is characterized by relatively high concentrations of photosynthetic pigments (Fig. 3, Table 5). It also has been observed that under low light conditions, the relative concentration of phycobillins increases (e.g., Millie et al. 1990; DeNobel et al. 1998; Jodłowska and Latała 2010) affecting the spectral shape of absorption (e.g., Stramski and Morel 1990; Berberoglu and Pilon 2007; Wojtasiewicz and Stramski 2010).

**Table 9:** Comparison between the values of chlorophyll-specific absorption coefficients *a*_p_*(440) and *a*_p_*(675) for cyanobacteria monocultures grown under various light conditions (values marked with *italics* obtained by digitizing appropriate figures in cited publications)

	Sample type	Light intensity	*a*_p_*(λ) (m^2^ mg Chl_a_^−1^)	
			440 nm	675 nm
This study	Cyanobacteria monocultures	7 μmol photons m^−2^ s^−1^	0.017–0.065	0.014–0.042
(Wojtasiewicz and Stramski 2010)	Cyanobacteria monocultures	70 μmol photons m^−2^ s^−1^	0.032–0.052	0.020–0.031
(Dupouy et al. 2008)	*Trichodesmium* spp.	Natural	0.0278	0.0192
(Metsamaa et al. 2006)	*Aphanizomenon flos-aquae*	25 μmol photons m^−2^ s^−1^	*0.032*	*0.017*
	*Anabaena circinalis*		*0.031*	*0.016*
	*Nodularia spumigena*		*0.025*	*0.014*
(Subramaniam et al. 1999)	*Trichodesmium* spp.	Natural	*0.04*	*0.01*
(Ahn et al. 1992)	*Anacystis marina*	100 μmol photons m^−2^ s^−1^	*0.075*	*0.025*
	*Synechocystis* sp.		*0.085*	*0.021*
	*Synechococcus* sp.		*0.12*	*0.045*
(Stramski and Morel 1990)	*Synechocystis* sp.	20–1450 μmol photons m^−2^ s^−1^	0.04–0.18	–

It is also worth noting that a number of remote sensing algorithms has been created to detect harmful algal blooms in various kinds of water reservoirs (Craig et al. 2006; Reinart and Kutser 2006; Hu et al. 2010). However, in inland and coastal environments, the satellite signal is often highly affected by the presence of dissolved organic matter absorbing strongly the light from the short-wave part of the spectrum, which is not the case in the detection methods based on the phytoplankton optical features.
